# Volume electron microscopy in axon regeneration research: insights into mitochondria, endoplasmic reticulum, and membrane contacts

**DOI:** 10.3389/fncir.2026.1870424

**Published:** 2026-07-01

**Authors:** Hiromi Tamada

**Affiliations:** Department of Anatomy, Graduate School of Medical Sciences, University of Fukui, Fukui, Japan

**Keywords:** peripheral nerve injury, axon initial segment, mitochondria, endoplasmic reticulum, microglia, volume electron microscopy, focused ion beam/scanning electron microscopy

## Abstract

Analysing organelle structures in tissue samples that preserve the *in vivo* environment, rather than in isolated cells, can provide valuable information on the mechanisms underlying nerve recovery and repair. However, determining organelle ultrastructure within tissue samples remains challenging with conventional microscopic techniques. To address this limitation, volume electron microscopy, particularly focused ion beam/scanning electron microscopy (FIB/SEM), has provided novel insights into organelle morphology, distribution, and membrane contacts, in three-dimensions and even within intact tissues. This review highlights the application of FIB/SEM for exploring the three-dimensional organization of mitochondria in motor neuron cell bodies and along the axon initial segments (AIS), where simultaneous investigation of intracellular and extracellular environments is difficult using other approaches. These analyses have revealed novel findings regarding mitochondrial distribution under healthy conditions and its dramatic alteration following injury, as well as microglial attachment around the AIS. Furthermore, FIB/SEM has enabled detailed characterization of the complex endoplasmic reticulum (ER) architecture within motor neuron cell bodies. Three-dimensional reconstructions have demonstrated a distinct uneven distribution of the ER in healthy neurons and revealed disruption of this organization following injury. In addition, ER–plasma membrane (ER–PM) contacts have been characterized as sheet-like structures, and quantitative analyses have shown significant increases in ER–PM contacts after injury. The novel findings obtained through FIB/SEM provide new perspectives on the cellular mechanisms underlying neuroregeneration and highlight the value of volume electron microscopy in advancing our understanding of nerve repair processes.

## 1 Introduction

Peripheral nerve injury is well known to recover after injury, whereas recovery following central nervous system (CNS) injuries, such as spinal cord injury, remains limited (He and Jin, 2016). Understanding the mechanisms underlying peripheral nerve regeneration could provide valuable insights into approaches for promoting CNS neuroregeneration. In particular, surrounding factors, such as the extracellular matrix and immune cells, strongly influence nerve recovery processes. In this context, tissue-level analysis is important for understanding these mechanisms without cell isolation.

Organelle responses are crucial for nerve recovery. For example, mitochondria frequently undergo structural changes, such as fusion and fission, to regulate energy production, redox homeostasis, and cellular apoptosis (Charrasse et al., 2024; Youle and van der Bliek, 2012; Giacomello et al., 2020; Friedman and Nunnari, 2014; Preminger and Schuldiner, 2024). Disruption of these regulatory mechanisms can trigger several pathological conditions, including neurodegenerative diseases (Berman et al., 2009; Bilsland et al., 2010; Chan, 2012; Knott A. B. et al., 2008; Sirois et al., 2026). In particular, mitochondrial transport between the cell body and axon is required for nerve recovery; therefore, the mechanisms regulating mitochondrial transport and the accompanying morphological changes are important research targets in axon regeneration (Cho et al., 2009; Kiryu-Seo et al., 2010; Wang et al., 2021; Kiryu-Seo et al., 2016). The endoplasmic reticulum (ER) also exhibits a close relationship between morphology and function (Kors and Schlaitz, 2024; Friedman et al., 2010; Sawyer et al., 2024; Voeltz et al., 2006; Schroeder et al., 2019; Bragulat-Teixidor et al., 2024; Westrate et al., 2015; Puhka et al., 2012; Heinrich et al., 2021; Obara et al., 2023; Arruda and Parlakgül, 2023). The ER forms a continuous membrane network and plays essential roles in cellular homeostasis. Disruption of ER morphology, has been implicated in various diseases, highlighting the close relationship between ER structure and function (Westrate et al., 2015). Furthermore, membrane contact sites (MCSs) between the ER and other organelles, including mitochondria and the plasma membrane (PM), regulate crucial processes related to signal transduction and membrane dynamics (Miyazono et al., 2018). Disruption of MCSs has been associated with diverse neuronal pathologies (Watanabe et al., 2016; Saheki and De Camilli, 2017; Prinz et al., 2020).

One of the latest methodological trends in studying organelle morphology is fluorescence-based imaging with super-resolution microscopy (Nixon-Abell et al., 2016; Goujon et al., 2019; Schroeder et al., 2019). These methods provide a vast amount of information, particularly for isolated or cultured cells. However, for studying nerve recovery mechanisms, such samples may not fully reflect *in vivo* conditions because cell isolation dramatically alters the surrounding environment and may activate numerous physiological responses within the cells themselves. To obtain a more accurate understanding of neuroregeneration *in vivo*, electron microscopy (EM), particularly volume electron microscopy (vEM), is a highly suitable tool (Tamada, 2023), as it provides three-dimensional ultrastructural information without requiring isolation from surrounding tissues.

vEM (Denk and Horstmann, 2004; Merchan-Perez et al., 2009; Ohta et al., 2012; Hayworth et al., 2015; Narayan and Subramaniam, 2015; Xu et al., 2017; Peddie et al., 2022; Tamada, 2023), including focused ion beam/scanning electron microscopy (FIB/SEM) (Knott G. et al., 2008; Xu et al., 2021; Heinrich et al., 2021; Denk et al., 2012), the serial block face–SEM (SBF-SEM) (Denk and Horstmann, 2004; Wilke et al., 2013), and array tomography (Hayworth et al., 2015; Morgan et al., 2016), acquires multiple electron micrographs that are stacked to generate three-dimensional ultrastructural reconstructions. Among the techniques, FIB/SEM is particularly suitable for studying organelle architecture because its z-resolution, achieved through gallium ion beam milling, enables accurate reconstruction of sheet-like structures and membrane contact sites.

In this review, we focus on novel insights into mitochondria, the ER, and ER–PM contacts in mouse hypoglossal and sciatic motor neurons following peripheral nerve transection, a model of recoverable peripheral nerve injury, using FIB/SEM (Tamada et al., 2017; Tamada et al., 2021; Elgendy et al., 2024). vEM analysis of organelles overcomes the limitations of conventional transmission EM (TEM) analysis (Puhka et al., 2012; Terasaki et al., 2013; Zamponi et al., 2022), which provides only two-dimensional ultrastructural information. Consequently, vEM enables quantitative assessment of geometrical parameters, such as organelle size, number, and spatial distribution. Based on these three-dimensional findings, we discuss new perspectives on the mechanisms underlying neuroregeneration after injury.

## 2 vEM analysis of mitochondria in axon regeneration models

### 2.1 Mitochondrial distribution in the cell body and the AIS

As described in the previous section, mitochondria exhibit a variety of morphologies, including elongated, spherical, and branched forms, particularly within neuronal cell bodies. They also undergo dynamic morphological changes, termed fission and fusion, in response to cellular demands. Mitochondrial fragmentation following neuronal injury has been extensively studied in axons and is considered crucial for the efficient and rapid delivery of energy and Ca^2+^ buffering capacity to regenerating axon tips (Cho et al., 2009; Kiryu-Seo et al., 2010; Wang et al., 2021; Kiryu-Seo et al., 2016). Although pathologically enlarged mitochondria (Knott A. B. et al., 2008; Waterham et al., 2007; Kiryu-Seo et al., 2016) and sparsely distributed mitochondria in axons have been observed by live-cell light microscopy (Kiryu-Seo et al., 2016; Tosolini et al., 2024) and electron tomography (Hoffmann et al., 2021), the numerous mitochondria present in neuronal somata and those of normal size generally require EM for detailed visualization. Furthermore, accurate assessment of mitochondrial morphology and distribution, including fragmentation status and density, requires three-dimensional ultrastructural analysis using vEM.

The quantitative data from FIB/SEM analysis revealed no significant differences in mitochondrial morphology or size within neuronal cell bodies following injury when intact and axon-injured neurons were compared (Tamada et al., 2017). Because these findings differed from the expectation that mitochondrial fragmentation would be prominent in injured neurons, attention shifted to another region, the axon initial segment (AIS), which is morphologically defined as the region between the axon hillock and the first myelinated segment. The AIS is a highly specialized domain characterized by distinct cytoskeletal organization and the expression of specific membrane channels and receptors that function in action potential initiation and as a boundary between the cell body and axonal compartments (Rasband, 2010; Kuba et al., 2010; Ogawa and Rasband, 2008; Garrido et al., 2003; Pan et al., 2006; Zhou et al., 1998; Leterrier, 2018; Nelson and Jenkins, 2017; Garrido, 2026). Although some TEM studies have reported the presence of mitochondria within the AIS (Dimova and Markov, 1976; Li et al., 2004; Sasaki et al., 2005), single ultrathin sections are insufficient to accurately determine their distribution because the AIS extends for approximately 25 μm. FIB/SEM analysis enables visualization of entire mitochondrial structures and their distribution throughout the full length of the AIS (Tamada et al., 2021).

**Figure 1:**
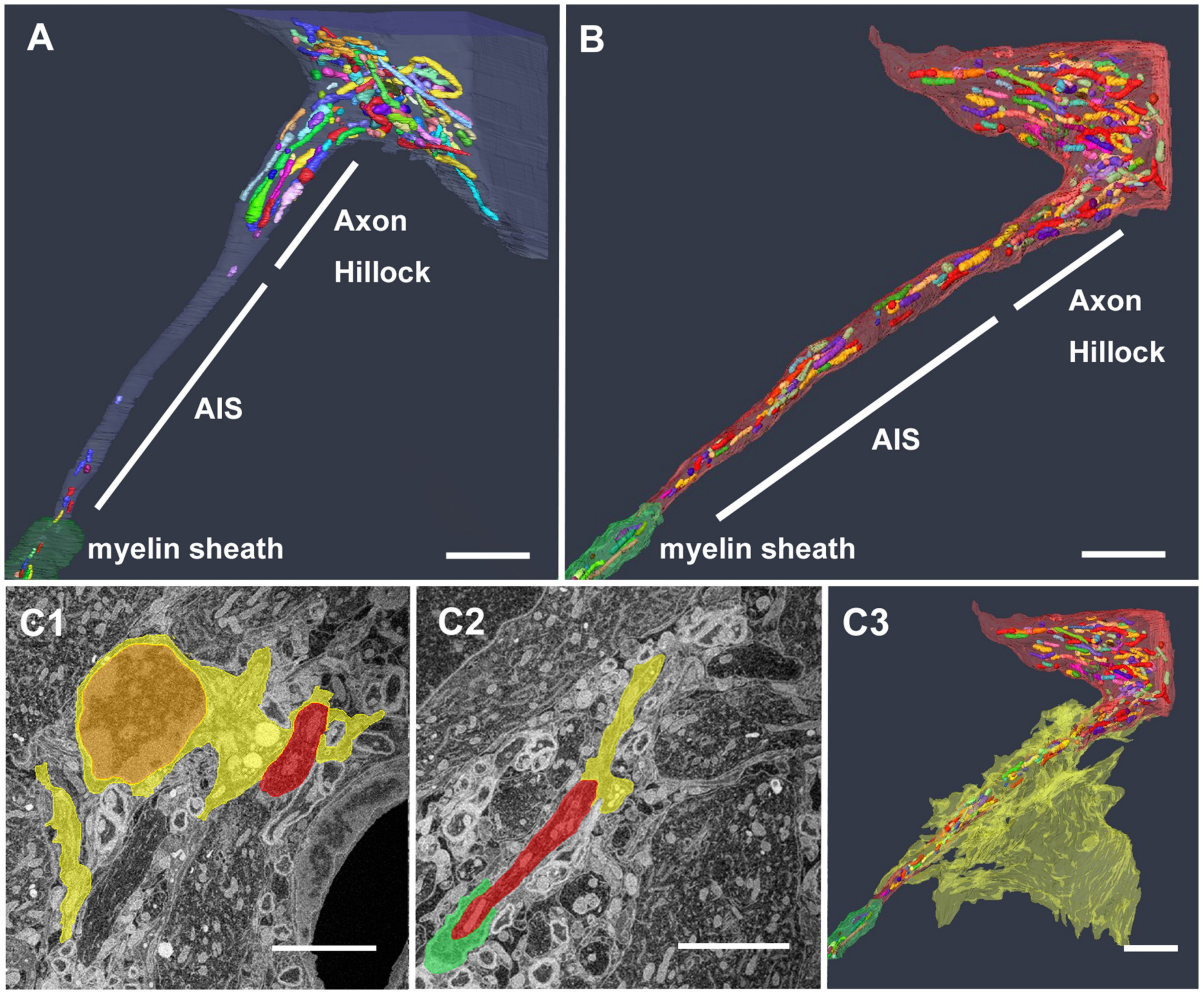
**(A)** Characteristic mitochondrial distribution around the axon initial segment (AIS) in healthy motor neurons. Mitochondria were observed in the soma, axon hillock, and myelinated regions (green). In contrast, mitochondria were largely absent from the AIS. **(B)** Altered mitochondrial distribution in the AIS following injury. In injured motor neurons, numerous mitochondria were distributed throughout the AIS. Their distribution pattern was similar to that observed in the soma, axon hillock, and myelinated regions. **(C)** Microglial association with the AIS following injury, corresponding to the same region shown in panel **B**. The **C1** and **C2** panels show representative FIB/SEM images demonstrating the absence of intervening cellular elements between the AIS and microglia. The **C3** panel shows a three-dimensional reconstruction of the soma, AIS, microglia, and surrounding cells. Yellow: microglia. Scale bar 5 μm. **A**, **B**, **C1** and **C2** are modified from Tamada et al. (2021).

Surprisingly, FIB/SEM demonstrated that healthy motor neurons contain almost no mitochondria within the AIS, despite the abundance of mitochondria in the axon hillock, similar to that observed in the soma, and their reappearance in the first myelinated region (Figure 1A) (Tamada et al., 2021). In contrast, following axonal injury, large numbers of mitochondria were present within the AIS at densities comparable to those observed in other neuronal regions (Figure 1B). In parallel, FIB/SEM analysis revealed direct attachment of microglia to the AIS membrane without the interposition of other cellular elements (Figure 1C) (Tamada et al., 2021). These findings suggest that the membrane environment of the AIS changes following injury and becomes more similar to that of the neuronal cell body, as the specialized membrane organization of the healthy AIS, including its unique channels and scaffolding proteins, may normally restrict cellular adhesion.

### 2.2 Interpretation of the findings

Using FIB/SEM analysis of the soma in peripherally injured motor neurons, a predominance of fragmented mitochondria could not be detected, which was one of the notable findings of this study. To date, the critical position of mitochondrial fragmentation during axonal repair has not been fully elucidated. Recent studies have suggested that the balance between mitochondrial fusion and fission, rather than fragmentation alone, is important for effective mitochondrial transport within axons (Au et al., 2022).

The marked alterations in mitochondrial distribution, from near exclusive in the healthy AIS to substantial accumulation following injury, are particularly noteworthy. One possible explanation for this influx of mitochondria is the disruption of the specialized molecular organization of the AIS, as indicated by microglial reactions and depletion of the cytoskeletal protein ankyrin G (AnkG) following injury (Tamada et al., 2021). Injury-induced degradation of AnkG has been associated with the loss of neuronal polarity and the expression of microtubule-associated protein (MAP2), a dendritic marker (Schafer et al., 2009). Under these conditions, somatodendritic proteins and organelles can enter the axon because of impaired polarity maintenance (Hedstrom et al., 2008; Teliska et al., 2022). Recently, similar phenomena, including mitochondrial exclusion from the healthy AIS and mitochondrial accumulation within the AIS under conditions of AIS disruption, have been reported in both *Drosophila* neurons (Wodrich et al., 2024) and human iPSC-derived neurons (Tjiang and Zempel, 2022). Although the mechanisms responsible for mitochondrial exclusion from the healthy AIS remain unclear (Yang et al., 2023), the accumulation of mitochondria under pathological conditions may reflect a protective cellular response.

However, mitochondria are continuously transported between the cell body and axon even under healthy conditions. Therefore, the differences observed between healthy and injured neurons may reflect changes not in mitochondrial “motility” itself but in the proportion of “stationary” mitochondria within the AIS. If the mitochondria accumulated in the injured AIS are predominantly stationary, they may contribute to buffering cytosolic Ca^2+^ levels, which are likely elevated following injury. The relationship between increased intracellular Ca^2+^ levels and AnkG degradation has been well documented and involves calpain, a calcium-dependent cysteine protease (Ma, 2013; Schafer et al., 2009; Del Puerto et al., 2015; Varadarajan et al., 2022). Furthermore, Benusa et al. (2017) described a mechanism by which microglia surrounding the AIS induce Ca^2+^ upregulation and activity, accompanied by calpain-dependent AnkG degradation. The presence of microglia around the AIS, as revealed by FIB/SEM analysis, is consistent with this hypothesis. Additional studies have reported that increased microglial activity influences the AIS under injury and inflammatory conditions, including those observed in axotomy models (Clark et al., 2016; Kato et al., 2016; Li et al., 2012). Although the functional significance of microglial contacts with the AIS remains controversial, differences in microglial subtypes and the roles of AIS-generated action potentials in regulating neuronal function may contribute to these varying observations (Baalman et al., 2015; Gallo et al., 2022; Que et al., 2024; Jenkins and Bender, 2025).

## 3 vEM analysis of ER and ER–PM contacts in axon regeneration models

### 3.1 Morphological findings of ER and ER–PM contact alterations after injury

The complex organization of the ER makes it difficult for light microscopy to resolve detailed membrane structures, particularly within neuronal cell bodies. Most current knowledge regarding pathological alterations in ER morphology and ER–PM contacts has been derived from isolated cultured mammalian cells, *C. elegans*, yeast, and other model systems, in which extensive ER stacking is rarely observed (Calì and Brini, 2021; Nakatsu and Tsukiji, 2023; Gamuyao and Chang, 2024). As described below, FIB/SEM analysis has provided novel three-dimensional insights into ER architecture and distribution while preserving native cell–cell interactions (Bharathan et al., 2023).

**Figure 2:**
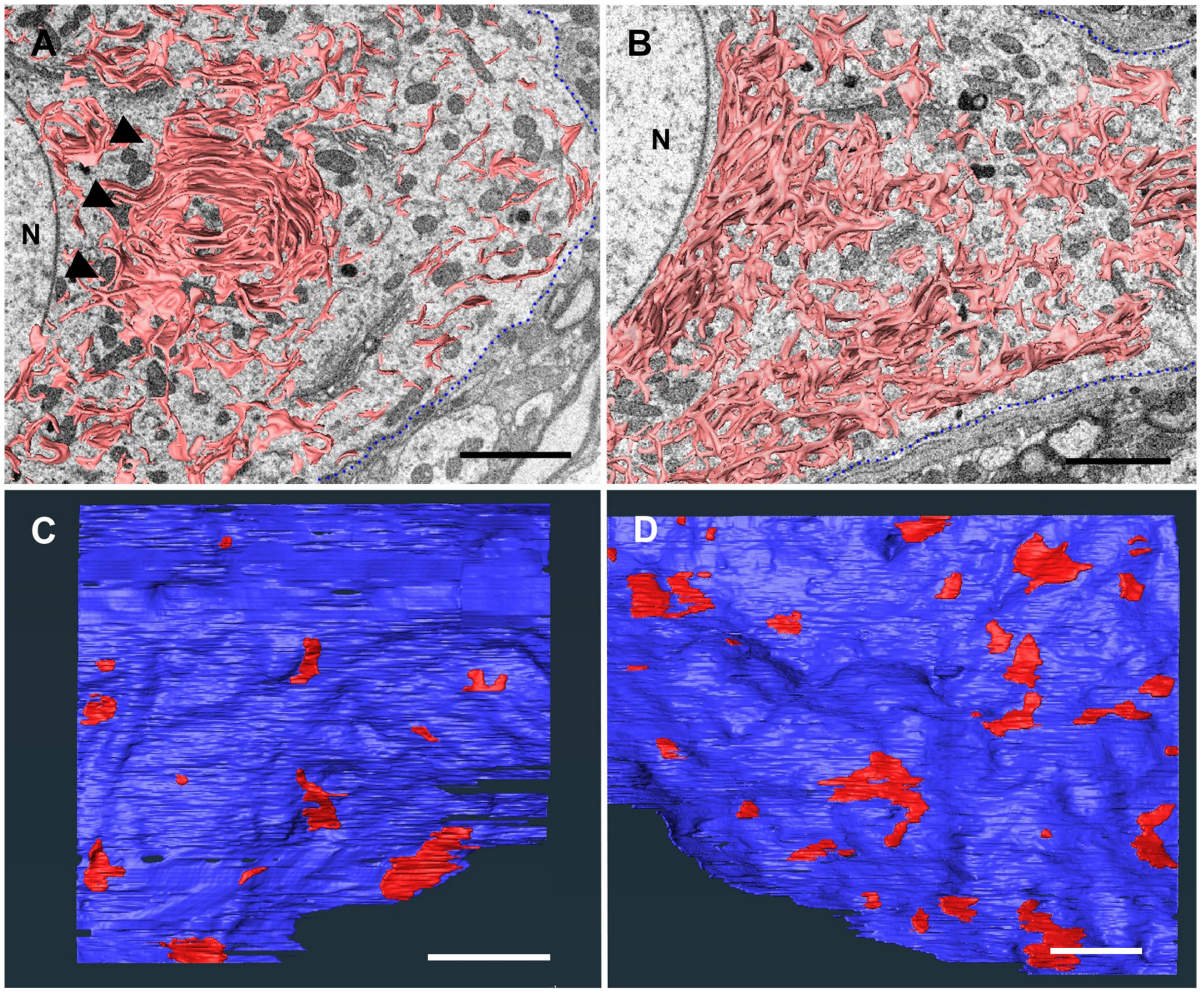
**(A)** Representative three-dimensional reconstruction of a motor neuron cell body. ER sheets (arrowheads) were abundant in the perinuclear region, whereas sparse ER tubules were observed in the peripheral region. Plasma membrane (PM) is indicated by blue dotted lines. N, nucleus. **(B)** Representative three-dimensional reconstruction of an injured motor neuron. The ER sheet structure was less distinct in the perinuclear region, and the ER was distributed more uniformly regardless of its proximity to the nucleus or PM (blue dotted lines). N, nucleus. **(C)** Three-dimensional reconstruction of ER–PM contacts in the motor neuron cell body. Red areas indicate individual ER–PM contact sites. The ER was attached to the PM (blue) in a patch-like manner. **(D)** ER–PM contacts in an injured motor neuron. Red sheets indicate ER–PM contact sites. Their surface areas were larger than those observed in healthy motor neurons (panel **C**). PM, blue. Scale bar 2 μm. These images are modified from Elgendy et al. (2024).

In the cell bodies of healthy motor neurons, FIB/SEM revealed an uneven ER distribution between the perinuclear region and the cell periphery near the plasma membrane (Figure 2A) (Elgendy et al., 2024). The ER surrounding the nucleus exhibited a lamellar structure, which is thought to correspond to Nissl bodies (tigroid substance). In contrast, the peripheral ER displayed a mesh-like organization, with a significantly smaller volume fraction than that observed in the perinuclear region. Following injury, these lamellar structures collapsed and were replaced by a more uniformly distributed mesh-like network (Figure 2B) (Elgendy et al., 2024). These qualitative changes were further supported by quantitative analyses of branching points associated with mesh formation.

Simultaneously, vEM analyses clearly visualized ER–PM contacts as sheet-like structures, enabling quantitative measurement of their surface area (Wu et al., 2017). ER–PM junctions vary substantially among cell types and physiological states with respect to abundance, molecular composition, and the extent of membrane apposition to the PM (Chang et al., 2017; Chung et al., 2022; Deardorff et al., 2014; Wu et al., 2017). Analysis of the hypoglossal axotomy model demonstrated that ER–PM contacts increased significantly, from approximately 3% in healthy motor neurons to 7% 1 week after injury (Figures 2C,D), indicating a marked enhancement of ER–PM interactions during the recovery process (Elgendy et al., 2024).

### 3.2 Interpretation of the findings

ER stress occurs under various forms of cellular stress, including axonal injury, and numerous studies have reported a close relationship between ER stress and alterations in ER morphology (Walter and Ron, 2011; Hetz and Saxena, 2017; Oñate et al., 2016; Wang and Kaufman, 2016; Öztürk et al., 2020). In the context of regenerative responses following axotomy, ER remodelling may play important roles not only at sites of axonal elongation but also within neuronal cell bodies (Lee et al., 2019; Yang et al., 2020; Carvalhais et al., 2026). Morphological alteration from a sheet-based to a tube-based structure may function as a resilience factor in hazardous situations by facilitating the transfer of intracellular molecules (Elgendy et al., 2024).

Several hypotheses may explain the increase in ER–PM contacts following injury. First, rapid and efficient lipid exchange between the ER and PM may be required to support membrane repair and replenishment (Guillén-Samander and De Camilli, 2023). In the hypoglossal axotomy model, expression of extended synaptotagmin-1 (E-Syt1) was significantly increased during the recovery phase (Elgendy et al., 2024). E-Syt1 is an important ER–PM tethering protein involved in lipid transfer between the ER and PM (Giordano et al., 2013; Jeyasimman and Saheki, 2020; Reinisch and De Camilli, 2016; Toulmay and Prinz, 2012; Saheki et al., 2016). In yeast, several stress conditions induce non-vesicular lipid transfer from the ER to the PM through regulation of tricalbin, the orthologue of E-Syt proteins (Thomas et al., 2022; Mu et al., 2025). Furthermore, previous studies suggest that the ER contributes membrane components required for axonal elongation (Rao et al., 2016; Zamponi et al., 2022), although information regarding similar mechanisms within neuronal cell bodies remains limited. A second possibility is that elevated intracellular Ca^2+^ levels contribute to the increase in ER–PM contacts. As discussed in the mitochondrial section of this review, intracellular Ca^2+^ concentrations may increase following injury. E-Syt1 is strongly regulated by Ca^2+^ because its C2 domains, which mediate interactions with the plasma membrane phosphatidylinositol 4,5-bisphosphate (PI(4,5)P₂), are activated by elevated cytosolic Ca^2+^ concentrations (Giordano et al., 2013; Jeyasimman and Saheki, 2020; Reinisch and De Camilli, 2016; Saheki et al., 2016).

From the perspective of Ca^2+^ regulation, quantitative geometric information regarding ER architecture, including the distance between the ER and PM, represents a particularly promising area for future vEM studies. Such parameters are likely to be important because Ca^2+^ signalling is highly dependent on the spatial relationships between Ca^2+^ sources, receptors, and Ca^2+^-activated ion channels (Tamada and Iino, 2025; Denizot et al., 2026; Tamada, 2026).

## 4 Future perspectives and current limitations

Although this review focused on mitochondria and the ER, numerous other factors contribute to the regulation of axonal structure and function (Smith et al., 2023). In particular, microtubules play essential roles in intracellular transport and organelle morphology. vEM has the potential to simultaneously visualize and analyse these diverse cellular components within their native tissue environment. However, for some targets, the effects of fixative artifacts resulting from conventional aldehyde-based fixation methods must be considered, as these procedures may alter ultrastructural features. As an alternative approach, cryofixation using high-pressure freezing can be employed (Tamada et al., 2020), although this method is not applicable to all sample types.

One of the current advances in vEM methodology is the development of correlative light and electron microscopy (CLEM) (Müller-Reichert and Verkade, 2012). This approach integrates information on protein localization and gene expression obtained by light microscopy with ultrastructural data acquired by EM, enabling a more comprehensive understanding of the relationships between cellular function and morphology (Ohta et al., 2021; Hayashi et al., 2023). Furthermore, rapid progress in deep-learning-based image analysis is expected to accelerate vEM research. Recent algorithms can generate near-isotropic image datasets from anisotropic data through denoising and super-resolution techniques (Lu et al., 2024). In addition, advances in automated image segmentation have the potential to reduce one of the major bottlenecks in vEM analysis, namely the burden of segmentation for three-dimensional structures from serial image datasets (Conrad and Narayan, 2023; Müller et al., 2024). Together, these technological developments are expected to generate large-scale datasets linking cellular ultrastructure with molecular and functional information, thereby advancing our understanding of neural regeneration mechanisms.

## 5 Conclusion

Recent advances in vEM have enabled three-dimensional ultrastructural analysis of tissue samples at high resolution. Using this approach, previously unrecognized patterns of mitochondrial and ER morphology and distribution have been identified in healthy motor neurons, as well as substantial alterations following injury in the context of surrounding cellular elements. Such observations are particularly important for understanding nerve recovery mechanisms because intracellular and extracellular factors interact in a highly coordinated manner during regeneration. These novel morphological insights complement conventional molecular and cellular approaches and provide new opportunities to advance the study of neuroregeneration.

## References

[ref1] Arruda A. P. Parlakgül G. (2023). Endoplasmic reticulum architecture and inter-organelle communication in metabolic health and disease. Cold Spring Harb. Perspect. Biol. 15:a041261. doi: 10.1101/cshperspect.a041261, 35940911 PMC9899651

[ref2] Au N. P. B. Chand R. Kumar G. Asthana P. Tam W. Y. Tang K. M. . (2022). A small molecule M1 promotes optic nerve regeneration to restore target-specific neural activity and visual function. Proc. Natl. Acad. Sci. USA 119:e2121273119. doi: 10.1073/pnas.2121273119, 36306327 PMC9636930

[ref3] Baalman K. Marin M. A. Ho T. S. Godoy M. Cherian L. Robertson C. . (2015). Axon initial segment-associated microglia. J. Neurosci. 35, 2283–2292. doi: 10.1523/JNEUROSCI.3751-14.2015, 25653382 PMC4315845

[ref4] Benusa S. D. George N. M. Sword B. A. DeVries G. H. Dupree J. L. (2017). Acute neuroinflammation induces AIS structural plasticity in a NOX2-dependent manner. J. Neuroinflammation 14:116. doi: 10.1186/s12974-017-0889-3, 28595650 PMC5465457

[ref5] Berman S. B. Chen Y. B. Qi B. McCaffery J. M. Rucker E. B. III Goebbels S. . (2009). Bcl-xL increases mitochondrial fission, fusion, and biomass in neurons. J. Cell Biol. 184, 707–719. doi: 10.1083/jcb.200809060, 19255249 PMC2686401

[ref6] Bharathan N. K. Giang W. Hoffman C. L. Aaron J. S. Khuon S. Chew T. L. . (2023). Architecture and dynamics of a desmosome-endoplasmic reticulum complex. Nat. Cell Biol. 25, 823–835. doi: 10.1038/s41556-023-01154-4, 37291267 PMC10960982

[ref7] Bilsland L. G. Sahai E. Kelly G. Golding M. Greensmith L. Schiavo G. (2010). Deficits in axonal transport precede ALS symptoms in vivo. Proc. Natl. Acad. Sci. USA 107, 20523–20528. doi: 10.1073/pnas.1006869107, 21059924 PMC2996651

[ref8] Bragulat-Teixidor H. Ishihara K. Szücs G. M. Otsuka S. (2024). The endoplasmic reticulum connects to the nucleus by constricted junctions that mature after mitosis. EMBO Rep. 25, 3137–3159. doi: 10.1038/s44319-024-00175-w, 38877171 PMC11239909

[ref9] Calì T. Brini M. (2021). Quantification of organelle contact sites by split-GFP-based contact site sensors (SPLICS) in living cells. Nat. Protoc. 16, 5287–5308. doi: 10.1038/s41596-021-00614-1, 34686857

[ref10] Carvalhais L. G. Kole K. Kuijpers M. (2026). The diverse forms and roles of the neuronal endoplasmic reticulum. Nat. Rev. Neurosci. 27, 260–277. doi: 10.1038/s41583-025-01016-y, 41530523

[ref11] Chan D. C. (2012). Fusion and fission: interlinked processes critical for mitochondrial health. Annu. Rev. Genet. 46, 265–287. doi: 10.1146/annurev-genet-110410-132529, 22934639

[ref12] Chang C. L. Chen Y. J. Liou J. (2017). ER-plasma membrane junctions: why and how do we study them? Biochim. Biophys. Acta, Mol. Cell Res. 1864, 1494–1506. doi: 10.1016/j.bbamcr.2017.05.018, 28554772 PMC5542405

[ref13] Charrasse S. Racine V. Saint-Omer C. Poquillon T. Lionnard L. Ledru M. . (2024). Quantitative imaging and semiotic phenotyping of mitochondrial network morphology in live human cells. PLoS One 19:e0301372. doi: 10.1371/journal.pone.0301372, 38547143 PMC10977735

[ref14] Cho D. H. Nakamura T. Fang J. Cieplak P. Godzik A. Gu Z. . (2009). S-nitrosylation of Drp1 mediates beta-amyloid-related mitochondrial fission and neuronal injury. Science 324, 102–105. doi: 10.1126/science.1171091, 19342591 PMC2823371

[ref15] Chung G. H. C. Lorvellec M. Gissen P. Pichaud F. Burden J. J. Stefan C. J. (2022). The ultrastructural organization of endoplasmic reticulum-plasma membrane contacts is conserved in epithelial cells. Mol. Biol. Cell 33:ar113. doi: 10.1091/mbc.E21-11-0534-T, 35947498 PMC9635291

[ref16] Clark K. C. Josephson A. Benusa S. D. Hartley R. K. Baer M. Thummala S. . (2016). Compromised axon initial segment integrity in EAE is preceded by microglial reactivity and contact. Glia 64, 1190–1209. doi: 10.1002/glia.22991, 27100937

[ref17] Conrad R. Narayan K. (2023). Instance segmentation of mitochondria in electron microscopy images with a generalist deep learning model trained on a diverse dataset. Cell Syst. 14, 58–71.e5. doi: 10.1016/j.cels.2022.12.006, 36657391 PMC9883049

[ref18] Deardorff A. S. Romer S. H. Sonner P. M. Fyffe R. E. W. (2014). Swimming against the tide: investigations of the C-Bouton synapse. Front. Neural Circuits 8:106. doi: 10.3389/fncir.2014.00106, 25278842 PMC4167003

[ref19] Del Puerto A. Fronzaroli-Molinieres L. Perez-Alvarez M. J. Giraud P. Carlier E. Wandosell F. . (2015). ATP-P2X7 receptor modulates axon initial segment composition and function in physiological conditions and brain injury. Cereb. Cortex 25, 2282–2294. doi: 10.1093/cercor/bhu035, 24610121

[ref20] Denizot A. Castillo M. F. V. Puchenkov P. Calì C. De Schutter E. (2026). The ultrastructural properties of the endoplasmic reticulum govern microdomain signaling in perisynaptic astrocytic processes. Glia 74:e70091. doi: 10.1002/glia.70091, 41098061

[ref21] Denk W. Briggman K. L. Helmstaedter M. (2012). Structural neurobiology: missing link to a mechanistic understanding of neural computation. Nat. Rev. Neurosci. 13, 351–358. doi: 10.1038/nrn3169, 22353782

[ref22] Denk W. Horstmann H. (2004). Serial block-face scanning electron microscopy to reconstruct three-dimensional tissue nanostructure. PLoS Biol. 2:e329. doi: 10.1371/journal.pbio.0020329, 15514700 PMC524270

[ref23] Dimova R. N. Markov D. V. (1976). Hanges in the mitochondria in the initial part of the axon during regeneration. Acta Neuropathol. 36, 235–242. doi: 10.1007/BF00685367, 64104

[ref24] Elgendy M. Tamada H. Taira T. Iio Y. Kawamura A. Kunogi A. . (2024). Dynamic changes in endoplasmic reticulum morphology and its contact with the plasma membrane in motor neurons in response to nerve injury. Cell Tissue Res. 396, 71–84. doi: 10.1007/s00441-024-03858-x, 38311679 PMC10997708

[ref25] Friedman J. R. Nunnari J. (2014). Mitochondrial form and function. Nature 505, 335–343. doi: 10.1038/nature12985, 24429632 PMC4075653

[ref26] Friedman J. R. Webster B. M. Mastronarde D. N. Verhey K. J. Voeltz G. K. (2010). ER sliding dynamics and ER-mitochondrial contacts occur on acetylated microtubules. J. Cell Biol. 190, 363–375. doi: 10.1083/jcb.200911024, 20696706 PMC2922647

[ref27] Gallo N. B. Berisha A. Van Aelst L. (2022). Microglia regulate chandelier cell axo-axonic synaptogenesis. Proc. Natl. Acad. Sci. USA 119:e2114476119. doi: 10.1073/pnas.2114476119, 35263225 PMC8931231

[ref28] Gamuyao R. Chang C. L. (2024). Imaging and proteomics toolkits for studying organelle contact sites. Front. Cell Dev. Biol. 12:1466915. doi: 10.3389/fcell.2024.1466915, 39381373 PMC11458464

[ref29] Garrido J. J. (2026). Modulation of axon excitable domains by astrocytes and microglia. Front. Cell. Neurosci. 19:1749755. doi: 10.3389/fncel.2025.1749755, 41585446 PMC12823472

[ref30] Garrido J. J. Giraud P. Carlier E. Fernandes F. Moussif A. Fache M. P. . (2003). A targeting motif involved in sodium channel clustering at the axonal initial segment. Science 300, 2091–2094. doi: 10.1126/science.1085167, 12829783

[ref31] Giacomello M. Pyakurel A. Glytsou C. Scorrano L. (2020). The cell biology of mitochondrial membrane dynamics. Nat. Rev. Mol. Cell Biol. 21, 204–224. doi: 10.1038/s41580-020-0210-7, 32071438

[ref32] Giordano F. Saheki Y. Idevall-Hagren O. Colombo S. F. Pirruccello M. Milosevic I. . (2013). PI(4,5)P(2)-dependent and ca(2+)-regulated ER-PM interactions mediated by the extended synaptotagmins. Cell 153, 1494–1509. doi: 10.1016/j.cell.2013.05.026, 23791178 PMC3716012

[ref33] Goujon A. Colom A. Straková K. Mercier V. Mahecic D. Manley S. . (2019). Mechanosensitive fluorescent probes to image membrane tension in mitochondria, endoplasmic reticulum, and lysosomes. J. Am. Chem. Soc. 141, 3380–3384. doi: 10.1021/jacs.8b13189, 30744381

[ref34] Guillén-Samander A. De Camilli P. (2023). Endoplasmic reticulum membrane contact sites, lipid transport, and neurodegeneration. Cold Spring Harb. Perspect. Biol. 15:a041257. doi: 10.1101/cshperspect.a041257, 36123033 PMC10071438

[ref35] Hayashi S. Ohno N. Knott G. Molnár Z. (2023). Correlative light and volume electron microscopy to study brain development. Microscopy 72, 279–286. doi: 10.1093/jmicro/dfad002, 36620906

[ref36] Hayworth K. J. Xu C. S. Lu Z. Knott G. W. Fetter R. D. Tapia J. C. . (2015). Ultrastructurally smooth thick partitioning and volume stitching for large-scale connectomics. Nat. Methods 12, 319–322. doi: 10.1038/nmeth.3292, 25686390 PMC4382383

[ref37] He Z. Jin Y. (2016). Intrinsic control of axon regeneration. Neuron 90, 437–451. doi: 10.1016/j.neuron.2016.04.022, 27151637

[ref38] Hedstrom K. L. Ogawa Y. Rasband M. N. (2008). AnkyrinG is required for maintenance of the axon initial segment and neuronal polarity. J. Cell Biol. 183, 635–640. doi: 10.1083/jcb.200806112, 19001126 PMC2582894

[ref39] Heinrich L. Bennett D. Ackerman D. Park W. Bogovic J. Eckstein N. . (2021). Whole-cell organelle segmentation in volume electron microscopy. Nature 599, 141–146. doi: 10.1038/s41586-021-03977-3 34616042

[ref40] Hetz C. Saxena S. (2017). ER stress and the unfolded protein response in neurodegeneration. Nat. Rev. Neurosci. 13, 477–491. doi: 10.1038/nrneurol.2017.99, 28731040

[ref41] Hoffmann P. C. Giandomenico S. L. Ganeva I. Wozny M. R. Sutcliffe M. Lancaster M. A. . (2021). Electron cryo-tomography reveals the subcellular architecture of growing axons in human brain organoids. eLife 10:e70269. doi: 10.7554/eLife.70269, 34698018 PMC8547956

[ref42] Jenkins P. M. Bender K. J. (2025). Axon initial segment structure and function in health and disease. Physiol. Rev. 105, 765–801. doi: 10.1152/physrev.00030.2024, 39480263 PMC12239863

[ref43] Jeyasimman D. Saheki Y. (2020). SMP domain proteins in membrane lipid dynamics. Biochim. Biophys. Acta Mol. Cell Biol. Lipids 1865:158447. doi: 10.1016/j.bbalip.2019.04.007, 31002947

[ref44] Kato G. Inada H. Wake H. Akiyoshi R. Miyamoto A. Eto K. . (2016). Microglial contact prevents excess depolarization and rescues neurons from excitotoxicity. eNeuro 3:ENEURO.0004-16.2016. doi: 10.1523/ENEURO.0004-16.2016, 27390772 PMC4916329

[ref45] Kiryu-Seo S. Ohno N. Kidd G. J. Komuro H. Trapp B. D. (2010). Demyelination increases axonal stationary mitochondrial size and the speed of axonal mitochondrial transport. J. Neurosci. 30, 6658–6666. doi: 10.1523/JNEUROSCI.5265-09.2010, 20463228 PMC2885867

[ref46] Kiryu-Seo S. Tamada H. Kato Y. Yasuda K. Ishihara N. Nomura M. . (2016). Mitochondrial fission is an acute and adaptive response in injured motor neurons. Sci. Rep. 6:28331. doi: 10.1038/srep28331, 27319806 PMC4913268

[ref47] Knott G. Marchman H. Wall D. Lich B. (2008). Serial section scanning electron microscopy of adult brain tissue using focused ion beam milling. J. Neurosci. 28, 2959–2964. doi: 10.1523/JNEUROSCI.3189-07.2008, 18353998 PMC6670719

[ref48] Knott A. B. Perkins G. Schwarzenbacher R. Bossy-Wetzel E. (2008). Mitochondrial fragmentation in neurodegeneration. Nat. Rev. Neurosci. 9, 505–518. doi: 10.1038/nrn2417, 18568013 PMC2711514

[ref49] Kors S. Schlaitz A. L. (2024). Dynamic remodelling of the endoplasmic reticulum for mitosis. J. Cell Sci. 137:jcs261444. doi: 10.1242/jcs.261444, 39584405

[ref50] Kuba H. Oichi Y. Ohmori H. (2010). Presynaptic activity regulates Na(+) channel distribution at the axon initial segment. Nature 465, 1075–1078. doi: 10.1038/nature09087, 20543825

[ref52] Lee S. Wang W. Hwang J. Namgung U. Min K. T. (2019). Increased ER-mitochondria tethering promotes axon regeneration. Proc. Natl. Acad. Sci. USA 116, 16074–16079. doi: 10.1073/pnas.1818830116, 31332012 PMC6689909

[ref54] Leterrier C. (2018). The axon initial segment: an updated viewpoint. J. Neurosci. 38, 2135–2145. doi: 10.1523/JNEUROSCI.1922-17.2018, 29378864 PMC6596274

[ref55] Li Y. Du X. F. Liu C. S. Wen Z. L. Du J. L. (2012). Reciprocal regulation between resting microglial dynamics and neuronal activity in vivo. Dev. Cell 23, 1189–1202. doi: 10.1016/j.devcel.2012.10.027, 23201120

[ref56] Li Y. C. Zhai X. Y. Ohsato K. Futamata H. Shimada O. Atsumi S. (2004). Mitochondrial accumulation in the distal part of the initial segment of chicken spinal motoneurons. Brain Res. 1026, 235–243. doi: 10.1016/j.brainres.2004.08.016, 15488485

[ref57] Lu C. Chen K. Qiu H. Chen X. Chen G. Qi X. . (2024). Diffusion-based deep learning method for augmenting ultrastructural imaging and volume electron microscopy. Nat. Commun. 15:4677. doi: 10.1038/s41467-024-49125-z, 38824146 PMC11144272

[ref58] Ma M. (2013). Role of calpains in the injury-induced dysfunction and degeneration of the mammalian axon. Neurobiol. Dis. 60, 61–79. doi: 10.1016/j.nbd.2013.08.010, 23969238 PMC3882011

[ref59] Merchan-Perez A. Rodriguez J. R. Alonso-Nanclares L. Schertel A. Defelipe J. (2009). Counting synapses using FIB/SEM microscopy: a true revolution for ultrastructural volume reconstruction. Front. Neuroanat. 3:18. doi: 10.3389/neuro.05.018.2009, 19949485 PMC2784681

[ref60] Miyazono Y. Hirashima S. Ishihara N. Kusukawa J. Nakamura K. I. Ohta K. (2018). Uncoupled mitochondria quickly shorten along their long axis to form indented spheroids, instead of rings, in a fission-independent manner. Sci. Rep. 8:350. doi: 10.1038/s41598-017-18582-6, 29321618 PMC5762872

[ref61] Morgan J. L. Berger D. R. Wetzel A. W. Lichtman J. W. (2016). The fuzzy logic of network connectivity in mouse visual thalamus. Cell 165, 192–206. doi: 10.1016/j.cell.2016.02.033, 27015312 PMC4808248

[ref62] Mu B. Rutkowski D. M. Grenci G. Vavylonis D. Zhang D. (2025). Ca2+−dependent vesicular and non-vesicular lipid transfer controls hypoosmotic plasma membrane expansion. BMC Biol. 23:207. doi: 10.1186/s12915-025-02309-5, 40629316 PMC12239298

[ref63] Müller P. de la Cuesta-Zuluaga J. Kuhn M. Baghai Arassi M. Treis T. Blasche S. . (2024). High-throughput anaerobic screening for identifying compounds acting against gut bacteria in monocultures or communities. Nat. Protoc. 19, 668–699. doi: 10.1038/s41596-023-00926-4, 38092943

[ref64] Müller-Reichert T. Verkade P. (2012). Correlative Light and Electron Microscopy. London: Academic Press.

[ref65] Nakatsu F. Tsukiji S. (2023). Chemo- and opto-genetic tools for dissecting the role of membrane contact sites in living cells: recent advances and limitations. Curr. Opin. Chem. Biol. 73:102262. doi: 10.1016/j.cbpa.2022.102262, 36731242

[ref66] Narayan K. Subramaniam S. (2015). Focused ion beams in biology. Nat. Methods 12, 1021–1031. doi: 10.1038/nmeth.3623, 26513553 PMC6993138

[ref67] Nelson A. D. Jenkins P. M. (2017). Axonal membranes and their domains: assembly and function of the axon initial segment and node of Ranvier. Front. Cell. Neurosci. 11:136. doi: 10.3389/fncel.2017.00136, 28536506 PMC5422562

[ref68] Nixon-Abell J. Obara C. J. Weigel A. V. Li D. Legant W. R. Xu C. S. . (2016). Increased spatiotemporal resolution reveals highly dynamic dense tubular matrices in the peripheral ER. Science 354:aaf3928. doi: 10.1126/science.aaf3928, 27789813 PMC6528812

[ref69] Obara C. J. Moore A. S. Lippincott-Schwartz J. (2023). Structural diversity within the endoplasmic reticulum-from the microscale to the nanoscale. Cold Spring Harb. Perspect. Biol. 15:a041259. doi: 10.1101/cshperspect.a041259, 36123032 PMC10394098

[ref70] Ogawa Y. Rasband M. N. (2008). The functional organization and assembly of the axon initial segment. Curr. Opin. Neurobiol. 18, 307–313. doi: 10.1016/j.conb.2008.08.008, 18801432

[ref71] Ohta K. Hirashima S. Miyazono Y. Togo A. Nakamura K. I. (2021). Correlation of organelle dynamics between light microscopic live imaging and electron microscopic 3D architecture using FIB-SEM. Microscopy 70, 161–170. doi: 10.1093/jmicro/dfaa071, 33216938 PMC7989057

[ref72] Ohta K. Sadayama S. Togo A. Higashi R. Tanoue R. Nakamura K. (2012). Beam deceleration for block-face scanning electron microscopy of embedded biological tissue. Micron 43, 612–620. doi: 10.1016/j.micron.2011.11.001, 22285616

[ref73] Oñate M. Catenaccio A. Martinez G. Armentano D. Parsons G. Kerr B. . (2016). Activation of the unfolded protein response promotes axonal regeneration after peripheral nerve injury. Sci. Rep. 6:21709. doi: 10.1038/srep21709, 26906090 PMC4764858

[ref74] Öztürk Z. O’Kane C. J. Perez-Moreno J. J. (2020). Axonal endoplasmic reticulum dynamics and its roles in neurodegeneration. Front. Neurosci. 14:48. doi: 10.3389/fnins.2020.00048, 32116502 PMC7025499

[ref75] Pan Z. Kao T. Horvath Z. Lemos J. Sul J. Y. Cranstoun S. D. . (2006). A common ankyrin-G-based mechanism retains KCNQ and NaV channels at electrically active domains of the axon. J. Neurosci. 26, 2599–2613. doi: 10.1523/JNEUROSCI.4314-05.2006, 16525039 PMC6675151

[ref76] Peddie C. J. Genoud C. Kreshuk A. Meechan K. Micheva K. D. Narayan K. . (2022). Volume electron microscopy. Nat. Rev. Methods Primers 2:51. doi: 10.1038/s43586-022-00131-9, 37409324 PMC7614724

[ref77] Preminger N. Schuldiner M. (2024). Beyond fission and fusion-diving into the mysteries of mitochondrial shape. PLoS Biol. 22:e3002671. doi: 10.1371/journal.pbio.3002671, 38949997 PMC11216622

[ref78] Prinz W. A. Toulmay A. Balla T. (2020). The functional universe of membrane contact sites. Nat. Rev. Mol. Cell Biol. 21, 7–24. doi: 10.1038/s41580-019-0180-9, 31732717 PMC10619483

[ref79] Puhka M. Joensuu M. Vihinen H. Belevich I. Jokitalo E. (2012). Progressive sheet-to-tubule transformation is a general mechanism for endoplasmic reticulum partitioning in dividing mammalian cells. Mol. Biol. Cell 23, 2424–2432. doi: 10.1091/mbc.E10-12-0950, 22573885 PMC3386207

[ref80] Que Z. Olivero-Acosta M. I. Robinson M. Chen I. Zhang J. Wettschurack K. . (2024). Human iPSC-derived microglia sense and dampen hyperexcitability of cortical neurons carrying the epilepsy-associated SCN2A-L1342P mutation. J. Neurosci. 45:e2027232024. doi: 10.1523/JNEUROSCI.2027-23.2024, 39557580 PMC11735681

[ref81] Rao K. Stone M. C. Weiner A. T. Gheres K. W. Zhou C. Deitcher D. L. . (2016). Spastin, atlastin, and ER relocalization are involved in axon but not dendrite regeneration. Mol. Biol. Cell 27, 3245–3256. doi: 10.1091/mbc.E16-05-0287, 27605706 PMC5170858

[ref82] Rasband M. N. (2010). The axon initial segment and the maintenance of neuronal polarity. Nat. Rev. Neurosci. 11, 552–562. doi: 10.1038/nrn2852, 20631711

[ref83] Reinisch K. M. De Camilli P. (2016). SMP-domain proteins at membrane contact sites: structure and function. Biochim. Biophys. Acta 1861, 924–927. doi: 10.1016/j.bbalip.2015.12.003, 26686281 PMC4902782

[ref84] Saheki Y. Bian X. Schauder C. M. Sawaki Y. Surma M. A. Klose C. . (2016). Control of plasma membrane lipid homeostasis by the extended synaptotagmins. Nat. Cell Biol. 18, 504–515. doi: 10.1038/ncb3339, 27065097 PMC4848133

[ref85] Saheki Y. De Camilli P. (2017). Endoplasmic reticulum-plasma membrane contact sites. Annu. Rev. Biochem. 86, 659–684. doi: 10.1146/annurev-biochem-061516-044932, 28301744

[ref86] Sasaki S. Warita H. Abe K. Iwata M. (2005). Impairment of axonal transport in the axon hillock and the initial segment of anterior horn neurons in transgenic mice with a G93A mutant SOD1 gene. Acta Neuropathol. 110, 48–56. doi: 10.1007/s00401-005-1021-9, 15920660

[ref87] Sawyer E. M. Jensen L. E. Meehl J. B. Larsen K. P. Petito D. A. Hurley J. H. . (2024). SigmaR1 shapes rough endoplasmic reticulum membrane sheets. Dev. Cell 59, 2566–2577.e7. doi: 10.1016/j.devcel.2024.06.005, 38971154

[ref88] Schafer D. P. Jha S. Liu F. Akella T. McCullough L. D. Rasband M. N. (2009). Disruption of the axon initial segment cytoskeleton is a new mechanism for neuronal injury. J. Neurosci. 29, 13242–13254. doi: 10.1523/JNEUROSCI.3376-09.2009, 19846712 PMC2801423

[ref89] Schroeder L. K. Barentine A. E. S. Merta H. Schweighofer S. Zhang Y. Baddeley D. . (2019). Dynamic nanoscale morphology of the ER surveyed by STED microscopy. J. Cell Biol. 218, 83–96. doi: 10.1083/jcb.201809107, 30442642 PMC6314542

[ref90] Sirois C. L. Lee J. Chambers A. L. Zhao X. (2026). Mitochondrial dynamics in neurodevelopment and neurodevelopmental disorders. Nat. Rev. Neurosci. 27, 307–326. doi: 10.1038/s41583-026-01031-7, 41781679

[ref91] Smith G. Sweeney S. T. O'Kane C. J. Prokop A. (2023). How neurons maintain their axons long-term: an integrated view of axon biology and pathology. Front. Neurosci. 17:1236815. doi: 10.3389/fnins.2023.1236815, 37564364 PMC10410161

[ref92] Tamada H. (2023). Three-dimensional ultrastructure analysis of organelles in injured motor neuron. Anat. Sci. Int. 98, 360–369. doi: 10.1007/s12565-023-00720-y, 37071350 PMC10256651

[ref93] Tamada H. (2026). Novel volume-electron microscopic ultrastructural analysis of gastrointestinal excitability associated with calcium-activated chloride channels. J. Physiol. doi: 10.1113/JP287612 [Epud ahead of print]., 41965324

[ref94] Tamada H. Blanc J. Korogod N. Petersen C. C. Knott G. W. (2020). Ultrastructural comparison of dendritic spine morphology preserved with cryo and chemical fixation. eLife 9:e56384. doi: 10.7554/eLife.56384, 33274717 PMC7748412

[ref95] Tamada H. Iino S. (2025). ER morphological analysis associated with interstitial cells of Cajal and smooth muscle cells in the murine stomach. Cell Tissue Res. 402, 333–344. doi: 10.1007/s00441-025-04016-7, 41148282 PMC12727777

[ref96] Tamada H. Kiryu-Seo S. Hosokawa H. Ohta K. Ishihara N. Nomura M. . (2017). Three-dimensional analysis of somatic mitochondrial dynamics in fission-deficient injured motor neurons using FIB/SEM. J. Comp. Neurol. 525, 2535–2548. doi: 10.1002/cne.24213, 28324645

[ref97] Tamada H. Kiryu-Seo S. Sawada S. Kiyama H. (2021). Axonal injury alters the extracellular glial environment of the axon initial segment and allows substantial mitochondrial influx into axon initial segment. J. Comp. Neurol. 529, 3621–3632. doi: 10.1002/cne.25212, 34235750

[ref98] Teliska L. H. Dalla Costa I. Sert O. Twiss J. L. Rasband M. N. (2022). Axon initial segments are required for efficient motor neuron axon regeneration and functional recovery of synapses. J. Neurosci. 42, 8054–8065. doi: 10.1523/JNEUROSCI.1261-22.2022, 36096668 PMC9636994

[ref99] Terasaki M. Shemesh T. Kasthuri N. Klemm R. W. Schalek R. Hayworth K. J. . (2013). Stacked endoplasmic reticulum sheets are connected by helicoidal membrane motifs. Cell 154, 285–296. doi: 10.1016/j.cell.2013.06.031, 23870120 PMC3767119

[ref100] Thomas F. B. Omnus D. J. Bader J. M. Chung G. H. Kono N. Stefan C. J. (2022). Tricalbin proteins regulate plasma membrane phospholipid homeostasis. Life Sci. Alliance 5:e202201430. doi: 10.26508/lsa.202201430, 35440494 PMC9018018

[ref101] Tjiang N. Zempel H. (2022). A mitochondria cluster at the proximal axon initial segment controls axodendritic TAU trafficking in rodent primary and human iPSC-derived neurons. Cell. Mol. Life Sci. 79:120. doi: 10.1007/s00018-022-04150-3, 35119496 PMC8816743

[ref102] Tosolini A. P. Abatecola F. Negro S. Sleigh J. N. Schiavo G. (2024). The node of Ranvier influences the in vivo axonal transport of mitochondria and signaling endosomes. iScience 27:111158. doi: 10.1016/j.isci.2024.111158, 39524336 PMC11544082

[ref103] Toulmay A. Prinz W. A. (2012). A conserved membrane-binding domain targets proteins to organelle contact sites. J. Cell Sci. 125, 49–58. doi: 10.1242/jcs.085118, 22250200 PMC3269022

[ref104] Varadarajan S. Chumki S. A. Stephenson R. E. Misterovich E. R. Wu J. L. Dudley C. E. . (2022). Mechanosensitive calcium flashes promote sustained RhoA activation during tight junction remodeling. J. Cell Biol. 221:e202105107. doi: 10.1083/jcb.202105107, 35254388 PMC8906493

[ref105] Voeltz G. K. Prinz W. A. Shibata Y. Rist J. M. Rapoport T. A. (2006). A class of membrane proteins shaping the tubular endoplasmic reticulum. Cell 124, 573–586. doi: 10.1016/j.cell.2005.11.047, 16469703

[ref106] Walter P. Ron D. (2011). The unfolded protein response: from stress pathway to homeostatic regulation. Science 334, 1081–1086. doi: 10.1126/science.1209038, 22116877

[ref107] Wang B. Huang M. Shang D. Yan X. Zhao B. Zhang X. (2021). Mitochondrial behavior in axon degeneration and regeneration. Front. Aging Neurosci. 13:650038. doi: 10.3389/fnagi.2021.650038, 33762926 PMC7982458

[ref108] Wang M. Kaufman R. J. (2016). Protein misfolding in the endoplasmic reticulum as a conduit to human disease. Nature 529, 326–335. doi: 10.1038/nature17041, 26791723

[ref109] Watanabe S. Ilieva H. Tamada H. Nomura H. Komine O. Endo F. . (2016). Mitochondria-associated membrane collapse is a common pathomechanism in SIGMAR1- and SOD1-linked ALS. EMBO Mol. Med. 8, 1421–1437. doi: 10.15252/emmm.201606403, 27821430 PMC5167132

[ref110] Waterham H. R. Koster J. van Roermund C. W. Mooyer P. A. Wanders R. J. Leonard J. V. (2007). A lethal defect of mitochondrial and peroxisomal fission. EMBO Mol. Med. 356, 1736–1741. doi: 10.1056/NEJMoa064436, 17460227

[ref111] Westrate L. M. Lee J. E. Prinz W. A. Voeltz G. K. (2015). Form follows function: the importance of endoplasmic reticulum shape. Annu. Rev. Biochem. 84, 791–811. doi: 10.1146/annurev-biochem-072711-163501, 25580528

[ref112] Wilke S. A. Antonios J. K. Bushong E. A. Badkoobehi A. Malek E. Hwang M. . (2013). Deconstructing complexity: serial block-face electron microscopic analysis of the hippocampal mossy fiber synapse. J. Neurosci. 33, 507–522. doi: 10.1523/JNEUROSCI.1600-12.2013, 23303931 PMC3756657

[ref113] Wodrich A. P. K. Harris B. T. Giniger E. (2024). Changes in mitochondrial distribution occur at the axon initial segment in association with neurodegeneration in Drosophila. Biol. Open 13:bio060335. doi: 10.1242/bio.060335, 38912559 PMC11261633

[ref114] Wu Y. Whiteus C. Xu C. S. Hayworth K. J. Weinberg R. J. Hess H. F. . (2017). Contacts between the endoplasmic reticulum and other membranes in neurons. Proc. Natl. Acad. Sci. USA 114, E4859–E4867. doi: 10.1073/pnas.1701078114, 28559323 PMC5474793

[ref115] Xu C. S. Hayworth K. J. Lu Z. Grob P. Hassan A. M. Garcia-Cerdan J. G. . (2017). Enhanced FIB-SEM systems for large-volume 3D imaging. eLife 6:e25916. doi: 10.7554/eLife.25916, 28500755 PMC5476429

[ref116] Xu C. S. Pang S. Shtengel G. Müller A. Ritter A. T. Hoffman H. K. . (2021). An open-access volume electron microscopy atlas of whole cells and tissues. Nature 599, 147–151. doi: 10.1038/s41586-021-03992-4, 34616045 PMC9004664

[ref117] Yang S. Park J. H. Lu H. C. (2023). Axonal energy metabolism, and the effects in aging and neurodegenerative diseases. Mol. Neurodegener. 18:49. doi: 10.1186/s13024-023-00634-3, 37475056 PMC10357692

[ref118] Yang C. Wang X. Wang J. Wang X. Chen W. Lu N. . (2020). Rewiring neuronal glycerolipid metabolism determines the extent of axon regeneration. Neuron 105, 276–292.e5. doi: 10.1016/j.neuron.2019.10.009, 31786011 PMC6975164

[ref119] Youle R. J. van der Bliek A. M. (2012). Mitochondrial fission, fusion, and stress. Science 337, 1062–1065. doi: 10.1126/science.1219855, 22936770 PMC4762028

[ref120] Zamponi E. Meehl J. B. Voeltz G. K. (2022). The ER ladder is a unique morphological feature of developing mammalian axons. Dev. Cell 57, 1369–1382.e6. doi: 10.1016/j.devcel.2022.05.002, 35609616

[ref121] Zhou D. Lambert S. Malen P. L. Carpenter S. Boland L. M. Bennett V. (1998). AnkyrinG is required for clustering of voltage-gated Na channels at axon initial segments and for normal action potential firing. J. Cell Biol. 143, 1295–1304. doi: 10.1083/jcb.143.5.1295, 9832557 PMC2133082

